# An unusual cause of acute lower gastrointestinal bleeding: lung adenocarcinoma metastasis to the descending colon

**DOI:** 10.1002/ccr3.1039

**Published:** 2017-06-20

**Authors:** Ioannis S. Papanikolaou, Paraskevas Gkolfakis, Georgios Tziatzios, Konstantinos Grammatikos, Ioannis G. Panayiotides, Zoi Tsakiraki, Irina Zinovieva, George D. Dimitriadis, Konstantinos Triantafyllou

**Affiliations:** ^1^ Hepatogastroenterology Unit Second Department of Internal Medicine – Propaedeutic Research Institute and Diabetes Center Medical School National and Kapodistrian University of Athens “Attikon” University General Hospital Athens Greece; ^2^ 2nd Department of Pathology Medical School National and Kapodistrian University Attikon University General Hospital Athens Greece; ^3^ Department of Pathology Thriassion General Hospital of Eleusis Magoula Greece

**Keywords:** Adenocarcinoma, colon, descending, lung, metastasis

## Abstract

Lung adenocarcinoma with symptomatic GI metastasis occurs seldom in everyday clinical practice. However, as diagnostic modalities, therapeutic interventions, and supportive care for cancer evolve, it is likely that the clinician might encounter a number of similar cases in the future, and therefore, he should be aware of this rare entity.

## Question

**Figure 1:**
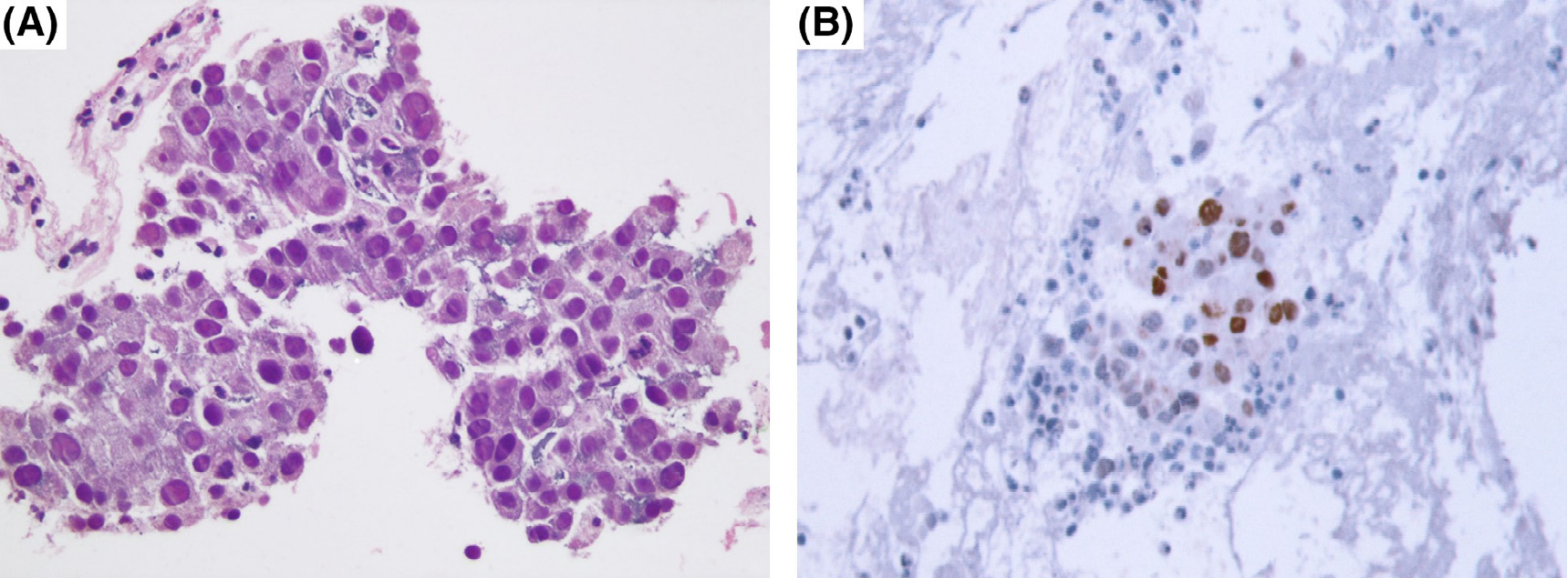
(A) Scant biopsy material disclosing a moderately differentiated adenocarcinoma (hematoxylin and eosin stain, x40), whose cells were immunopositive for TTF‐1(immunostain, x40) (B).

**Figure 2:**
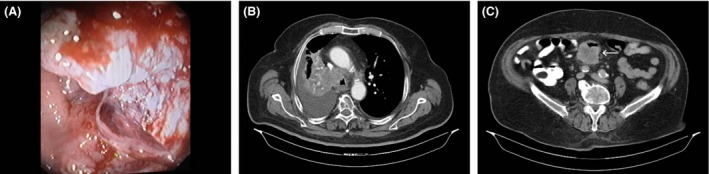
(A) Endoscopic view of the descending colon showing an ulcerated and bleeding lesion. (B) Chest computed tomography demonstrating the adenocarcinoma in the right upper pulmonary lobe. (C) Abdominal computed tomography showing an intraluminal mass in the descending colon (white arrow).

**Figure 3:**
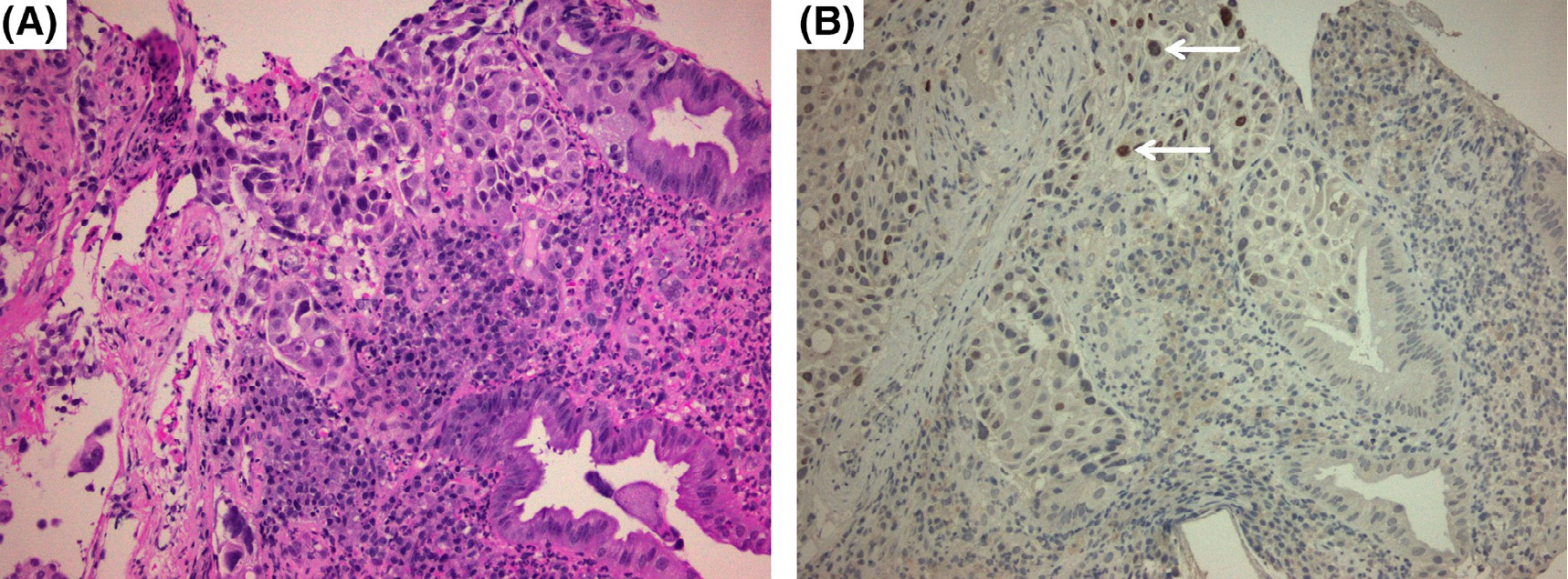
(A) An overview of colonic mucosa (glands) infiltrated by adenocarcinoma (hematoxylin and eosin stain, x20) with a few cells showing nuclear immunopositivity for TTF‐1 (brown staining; anti‐TTF‐1 immunostain, x20, white arrows) (B).

A 70‐year‐old Caucasian male patient was transferred to our department due to lower gastrointestinal bleeding. One year earlier, he had undergone a colonoscopy with no findings. At the same time, he had been diagnosed with right upper pulmonary lobe mass—histology confirmed lung adenocarcinoma (Fig. 1A and B). On admission, routine biochemical and hematological profiles were within normal ranges and the patient's clinical evaluation was unremarkable. Colonoscopy identified a bleeding descending colon lesion (Fig. 2A). Computed tomography of chest and abdomen showed the primary pulmonary mass (Fig. 2B) and the colonic lesion (Fig. 2C). Biopsy specimens obtained from the mass demonstrated a moderately differentiated adenocarcinoma (Fig. 3A). Immunostaining of tumor cells were positive for thyroid transcription factor‐1 (TTF‐1) and cytokeratin 7 (CK7), while negative for caudal‐related homeobox transcription factor 2 (CDX‐2) and cytokeratin 20 (CK‐20) (Fig. 3B), indicating a lung adenocarcinoma origin.

What is the diagnosis?

## Diagnosis

### Lung adenocarcinoma metastasis to the descending colon

Lung GI tract metastasis is an extremely rare event indicating diffuse metastatic disease and poor prognostic outcome 1.

At endoscopy, lung cancer metastasis to the GI tract cannot be distinguished from primary colorectal cancer because it has no specific macroscopic features. Histologic diagnosis is based upon morphologic differences between pulmonary and colonic adenocarcinomas (such as the so‐called dirty garland pattern frequently seen in the latter) and is corroborated by immunohistochemistry. TTF‐1 is the most frequently used immunohistochemical marker with a high specificity, both in tissue specimens and in pleural effusions. Combined TTF‐1 and CK7 immunopositivity is highly specific for the diagnosis of lung adenocarcinoma, while lack of CK20 and CDX2 rules out colonic type adenocarcinoma 2.

Lung adenocarcinoma patients commonly present with symptoms related to metastatic spread. Clinical presentation of colonic metastatic disease varies from asymptomatic patients to unspecific gastrointestinal symptoms 3. Meticulous investigation is mandatory when symptoms or signs implying GI tract involvement are present, prior to attributing them to cancer‐related (i.e., chemotherapy‐induced mucositis) causes.

## Informed Consent

Written informed consent was obtained from the patient for this case report.

## Conflict of Interest

All authors have no conflict of interest to declare.

## Authorship

ISP: revised the draft critically for important intellectual content and approved the manuscript. PG, GT, KG, ZT, and IZ: acquired the data, and drafted and approved the manuscript. IGP: analyzed and interpreted data, revised the draft critically for important intellectual content, and approved the manuscript. GD: revised the draft critically for important intellectual content and approved the manuscript. KT: conceived the idea, revised the draft critically for important intellectual content, and finally approved the manuscript.

## References

[1] Yoshimoto, A. , K. Kasahara , and A. Kawashima . 2006 Gastrointestinal metastases from primary lung cancer. Eur. J. Cancer 42:3157–3160. 17079136 10.1016/j.ejca.2006.08.030

[2] Gurda, G. T. , L. Zhang , Y. Wang , L. Chen , S. Geddes , W. C. Cho , et al. 2015 Utility of five commonly used immunohistochemical markers TTF‐1, Napsin A, CK7, CK5/6 and P63 in primary and metastatic adenocarcinoma and squamous cell carcinoma of the lung: a retrospective study of 246 fine needle aspiration cases. Clin. Transl. Med. 4:16. 25977750 10.1186/s40169-015-0057-2 PMC4417108

[3] Benedeto‐Stojanov, D. , G. Bjelaković , M. Milentijević , D. Stojanov , V. Brzački , and G. Petrović . 2014 Metastatic lesions in the gastroduodenum — an unusual manifestation of malignant melanoma and pulmonary adenocarcinoma. Cen. Eur. J. Med. 9:762–767.

